# Reduced Regional Brain Cortical Thickness in Patients with Heart Failure

**DOI:** 10.1371/journal.pone.0126595

**Published:** 2015-05-11

**Authors:** Rajesh Kumar, Santosh K. Yadav, Jose A. Palomares, Bumhee Park, Shantanu H. Joshi, Jennifer A. Ogren, Paul M. Macey, Gregg C. Fonarow, Ronald M. Harper, Mary A. Woo

**Affiliations:** 1 Department of Anesthesiology, University of California Los Angeles, Los Angeles, California, United States of America; 2 Department of Radiological Sciences, University of California Los Angeles, Los Angeles, California, United States of America; 3 Department of Bioengineering, University of California Los Angeles, Los Angeles, California, United States of America; 4 The Brain Research Institute, University of California Los Angeles, Los Angeles, California, United States of America; 5 Department of Neurology, University of California Los Angeles, Los Angeles, California, United States of America; 6 School of Nursing, University of California Los Angeles, Los Angeles, California, United States of America; 7 Division of Cardiology, University of California Los Angeles, Los Angeles, California, United States of America; 8 Department of Neurobiology, University of California Los Angeles, Los Angeles, California, United States of America

## Abstract

**Aims:**

Autonomic, cognitive, and neuropsychologic deficits appear in heart failure (HF) subjects, and these compromised functions depend on cerebral cortex integrity in addition to that of subcortical and brainstem sites. Impaired autoregulation, low cardiac output, sleep-disordered-breathing, hypertension, and diabetic conditions in HF offer considerable potential to affect cortical areas by loss of neurons and glia, which would be expressed as reduced cortical thicknesses. However, except for gross descriptions of cortical volume loss/injury, regional cortical thickness integrity in HF is unknown. Our goal was to assess regional cortical thicknesses across the brain in HF, compared to control subjects.

**Methods and Results:**

We examined localized cortical thicknesses in 35 HF and 61 control subjects with high-resolution T1-weighted images (3.0-Tesla MRI) using FreeSurfer software, and assessed group differences with analysis-of-covariance (covariates; age, gender; p<0.05; FDR). Significantly-reduced cortical thicknesses appeared in HF over controls in multiple areas, including the frontal, parietal, temporal, and occipital lobes, more markedly on the left side, within areas that control autonomic, cognitive, affective, language, and visual functions.

**Conclusion:**

Heart failure subjects show reduced regional cortical thicknesses in sites that control autonomic, cognitive, affective, language, and visual functions that are deficient in the condition. The findings suggest chronic tissue alterations, with regional changes reflecting loss of neurons and glia, and presumably are related to earlier-described axonal changes. The pathological mechanisms contributing to reduced cortical thicknesses likely include hypoxia/ischemia, accompanying impaired cerebral perfusion from reduced cardiac output and sleep-disordered-breathing and other comorbidities in HF.

## Introduction

Multiple autonomic, cognitive, and neuropsychologic deficits appear in heart failure (HF) subjects [1–3]. The impairments stem from structural brain changes in several gray matter sites in the forebrain and brainstem, white matter hyper-intensities, as well as interconnecting axons [4,5]. Those injured sites were detected by magnetic resonance imaging (MRI) procedures, including high-resolution T1-weighted imaging, T2-weighted imaging, T2-relaxometry, and diffusion tensor imaging (DTI) [4,6–8], which were accompanied by aberrant functional MRI responses to the Valsalva maneuver and cold pressor autonomic challenges [2,3]. Among the brain areas affected, cortical regions were apparent, especially areas that project to subcortical autonomic sites or influence cognitive and motor function.

Voxel-based MRI and visual examination procedures used to detect structural changes in HF, although useful for the first demonstrations of neural injury in the condition, have certain drawbacks, especially for cortical tissue evaluation. Visual examination procedures are poor for cortical injury detection over quantitative methods. However, both regional gray matter volumes and tissue integrity, assessed with voxel-based gray matter volumetric, T2-relaxometry, and DTI-based procedures, may be influenced by analytical differences in extra-cortical cerebrospinal fluid and surface curvature/complexities. An alternative approach is to examine regional cortical thicknesses, which can be performed with specialized software designed to assess regional cortical changes, and such assessment is provided by FreeSurfer software [9]. FreeSurfer analytical tools perform volumetric measurements with high-resolution T1-weighted images, using a spherical surface-based coordinate system that allows localizing brain sites with high accuracy, creating gray and white matter surfaces and pial boundaries, and allow for statistical assessment of regional cortical thicknesses [9]. Since FreeSurfer tools target the white matter surface for registration, gray matter tissue is not influenced by registration accuracy, and thus, the procedures may be useful to assess precisely regional cortical thicknesses in HF subjects.

Heart failure is accompanied by low cardiac output and sleep-disordered breathing [2,3,10]; both conditions may introduce hypoxic/ischemic-induced brain injury, affecting cortical sites with loss of neurons and supporting cells, resulting in reduced cortical thickness in areas especially sensitive to perfusion issues. Other comorbid conditions that may contribute to such regional cortical thinning in HF subjects include hypertension and diabetes. Regional cortical sites in HF have been examined grossly in a few studies [11–13], but specific brain regions with cortical thinning have not been assessed in the condition. Precise determination of reduced regional cortical thicknesses in sites that interact with subcortical and brainstem areas for appropriate actions may assist evaluation of processes underlying the deficient functional characteristics, including autonomic, mood, and cognitive issues, accurate cortical injury load, and potential development of Alzheimer’s disease in HF subjects.

Our aim was to examine regional cortical thicknesses across the whole brain in HF, compared to control subjects, using high-resolution T1-weighted images. We hypothesized that local cortical thinning would appear in HF over control subjects, especially in cortical areas, including the cingulate, insular, prefrontal, right middle frontal, hippocampus, and temporal sites serving autonomic, mood, and cognitive functions which are impaired in the condition.

## Materials and Methods

### Participants

We studied 35 hemodynamically-optimized HF and 61 control subjects. Demographic, clinical, body mass index (BMI), education, neuropsychologic, cognitive, and sleep data of HF and control subjects are summarized in Table 1. Since the majority of HF and control subjects were part of previously-published other studies [6,7,11], a 1:1 gender ratio could not be achieved. We included only HF and control subjects with ages between 30–65 years. The upper age limit was chosen to minimize age-related changes in sleep architecture and brain alterations, and the lower age limit to avoid developmental-related brain changes [14,15]. The diagnosis of HF was based on the left ventricular ejection fraction (LVEF < 0.40) and criteria from the American Heart Association [16], and all subjects included in this study were NYHA Functional Class II [17]. Of 35 HF subjects, 13 subjects were with ischemic and 22 with non-ischemic etiologies. Also, of 35 HF subjects, 18 subjects were hypertensive, 10 subjects had a history of diabetes, and none had a prior heart attack, history of substance abuse, including alcohol abuse, valvular congenital heart defects, or pregnancy induced cardiomyopathy. Heart failure subjects were recruited from the University of California at Los Angeles (UCLA) Cardiomyopathy Center and the Los Angeles area. All HF subjects underwent similar care and medications, were treated with angiotensin receptor blockers or angiotensin-converting enzyme inhibitors, beta blockers, and diuretics, and were stabilized for hemodynamics and body-weight for at least six months prior to participation in MRI studies. There medications and diuretic use were optimized based on pulmonary artery pressures and cardiac output. Control subjects were matched for the same age-range, but were not matched for BMI to HF subjects. All control subjects were recruited through advertisements at the UCLA campus and Los Angeles area, and were in good health, without any clinical history of cardiovascular, stroke, respiratory, renal dysfunction, drug abuse, traumatic brain injury, or neurologic and psychiatric conditions that might alter brain tissue. Both HF and control subjects were excluded from the study if they were claustrophobic, carrying non-removable metal, such as braces, embolic coils, pacemakers/implantable cardioverter-defibrillators, stents, or with body-weight more than 125 kg. HF and control subjects gave written and informed consent prior to participation in the study, and all procedures were approved by the Institutional Review Board at UCLA.

**Table 1:** Demographic, BMI, clinical, neuropsychologic, sleep, and cognitive variables of HF and control subjects.

Variables	HF	Controls	p values
Age (years)	54.9±8.5 (n = 35)	52.2±6.9 (n = 61)	0.10
Gender (male: female)	25:10 (n = 35)	40:21 (n = 61)	0.56
Handedness	Left, 2; Right, 31; Ambidextrous, 2	Left, 15; Right, 45; Ambidextrous, 1	0.043
BMI (kg/m^2^)	28.4±5.5 (n = 35)	25.4±3.5 (n = 61)	0.006
LVEF (%)	28.9±9.2 (n = 33)	-	-
Ethnicity	African American, 7; Asian, 4; Hispanic, 3; White, 20; Others, 1	African American, 3; Asian, 21; Hispanic, 6; White, 30; Others, 1	0.043
Education (years)	14.7±1.8 (n = 23)	17.6±3.8 (n = 23)	0.002
MoCA	24.2±3.5 (n = 15)	27.7±1.9 (n = 17)	0.002
T2D	11.4% (n = 35)		
BDI-II	10.3±7.1 (n = 27)	3.8±4.0 (n = 52)	<0.001
BAI	9.5±8.0 (n = 27)	4.2±5.2 (n = 52)	0.004
PSQI	7.2±3.9 (n = 27)	4.0±2.6 (n = 52)	<0.001
ESS	8.0±4.2 (n = 27)	5.2±3.2 (n = 52)	0.006

HF = Heart failure; BMI = Body-mass-index; LVEF = Left ventricular ejection fraction; MoCA = Montreal cognitive assessment; T2D = Type 2 diabetes; BDI-II = Beck depression inventory II; BAI = Beck anxiety inventory; PSQI = Pittsburgh sleep quality index; ESS, Epworth sleepiness scale.

### Data collection procedure

Both hemodynamically-optimized HF and control subjects were scanned largely before noon. All HF and control subjects were asked to refrain from caffeine, nicotine, and alcohol at least 24-hours prior to the study. Once subjects arrived at the MRI suite, we reviewed the study inclusion and exclusion criteria, explained the project, obtained written informed consent, and collected required information including age, gender, ethnicity, medications, measured height and weight, and introduced neuropsychologic, cognitive, and sleep questionnaires. All subjects were then taken to the MRI scanner room to become familiar with the scanner noise and equipment environment. Electrocardiogram, respiration, and blood oxygenation levels were monitored continuously to assure safety of HF and control subjects using MRI-compatible electrodes, air-filled bag for thoracic excursion, and a pulse oximeter. Subjects were positioned in the MRI scanner, and RK collected the required MRI data. Control and HF sample sizes were determined with the assumption of large effect sizes between groups, based on previous studies which included the majority of the same patient population [6,7,11].

### Sleep quality and daytime sleepiness examination

All HF and control subjects were assessed for sleep quality with the Pittsburgh sleep quality (PSQI), and daytime sleepiness with the Epworth sleepiness scale (ESS)[18,19]. The PSQI and ESS tests are self-administered questionnaires, which are commonly used indices of sleep quality and daytime sleepiness [18,19]. Both questionnaires were administered either immediately before or after MRI study.

### Assessment of depression and anxiety

Both depression and anxiety symptoms of HF and control subjects were assessed using the Beck depression inventory (BDI-II) and the Beck anxiety inventory (BAI), respectively [20–22]. The BDI-II and BAI inventories are self-administered questionnaires (21 questions; each score 0–3), with total scores ranging from 0–63 based on symptom severity [20–22]. Both questionnaires were administered either immediately before or after MRI examination.

### Cognitive examination

Cognitive deficits in HF and control subjects were examined with the Montreal Cognitive Assessment (MoCA) test [23], which is designed for rapid evaluation of cognitive domains, including attention and concentration, executive functions, memory, language, visuoconstructional skills, conceptual thinking, calculations, and orientation. A global MoCA score ≥26 is considered normal [23].

### Magnetic resonance imaging

We used a 3.0-Tesla MRI scanner (Siemens, Magnetom Tim-Trio, Erlangen, Germany) for the brain studies. All subjects lay supine during MRI scanning, and foam pads were used on both sides of the head to minimize head movement. Two high-resolution T1-weighted image volumes were acquired using a magnetization prepared rapid acquisition gradient-echo (MPRAGE) pulse sequence [repetition time (TR) = 2200 ms; echo-time (TE) = 2.34, 2.60 ms; inversion time = 900 ms; flip angle (FA) = 9°; matrix size = 256×256, 320 ×320; field of view (FOV) = 230×230 mm^2^; slice-thickness = 0.9, 1.0 mm]. We performed simultaneous proton-density (PD) and T2-weighted imaging (TR = 10,000 ms; TE1, 2 = 17, 134 ms; FA = 130°; matrix size = 256×256; FOV = 230×230 mm^2^; slice-thickness = 4.0 mm), covering the whole-brain in the axial plane using a dual-echo turbo spin-echo pulse sequence.

### Visual examination

Both high-resolution T1-weighted, PD-, and T2-weighted images were examined for any gross brain pathology, such as cysts, tumors, or any other mass lesions. None of the included HF and control subjects in this study showed any major brain pathology.

### Cortical thicknesses assessment

We used high-resolution T1-weighted images to measure regional cortical thicknesses in HF and control subjects using FreeSurfer (v 5.3.0) [9]. Both high-resolution T1-weighted image volumes, collected from each subject, were realigned and averaged to increase signal-to-noise ratio, and averaged brain volumes from each subject were converted into FreeSurfer data format (http://surfer.nmr.mgh.harvard.edu/). Data processing included removal of non-brain tissue using a hybrid watershed/surface deformation procedure, automated Talairach transformation, intensity normalization, segmentation of sub-cortical white and deep gray matter tissue types, tessellation of the gray and white matter boundaries, automated topology correction, and surface registration to the FreeSurfer atlas [9]. All subjects’ processed data were manually-evaluated by an investigator to ensure no brain areas were excluded. Similarly, gray, white, and pial boundaries were visually-assessed, and if needed, edits were made to correct misidentified regions. Such edits were required in a few HF and control subjects, which were performed by the same investigator who manually-evaluated all subjects.

### Statistical analysis

#### Demographic, BMI, neuropsychologic, sleep, cognitive, and clinical variables

The IBM statistical package for the social sciences (IBM SPSS, v 22, Chicago, IL) was used for data analyses. Demographic, BMI, neuropsychologic, sleep, cognitive, and clinical characteristics were assessed by independent samples t-tests and Chi-square. Relationships between BMI and regional cortical thicknesses were examined with Pearson’s correlation procedures. A p<0.05 value was considered statistically significant.

#### Regional cortical thicknesses

Gray matter surface maps were smoothed using a Gaussian kernel (full-width-at-half-maximum, 10 mm). Regional changes in cortical thicknesses between HF and control subjects were examined using a vertex-wise general linear model, implemented in FreeSurfer, with regional cortical thicknesses modeled as a function of groups, and age and gender included as covariates in the analysis (p<0.05, false discovery rate corrections for multiple comparisons). The statistical parametric maps with regional cortical thickness differences between HF and control groups were generated separately for left and right hemispheres. We overlaid clusters with significant differences between groups onto averaged inflated cortical surface maps for structural identification.

## Results

### Demographic, BMI, neuropsychologic, sleep, cognitive, and clinical variables

Demographic, BMI, neuropsychologic, sleep, cognitive, and clinical data of HF and control subjects are summarized in Table 1. No significant differences in age (p = 0.10) or gender (p = 0.55) appeared between groups. However, BMI (p = 0.006), BDI-II (p < 0.001), BAI (p = 0.004), PSQI (p < 0.001), ESS (p = 0.006), and MoCA (p = 0.002) values significantly differed between groups.

### Regional cortical thicknesses

**Fig 1:**
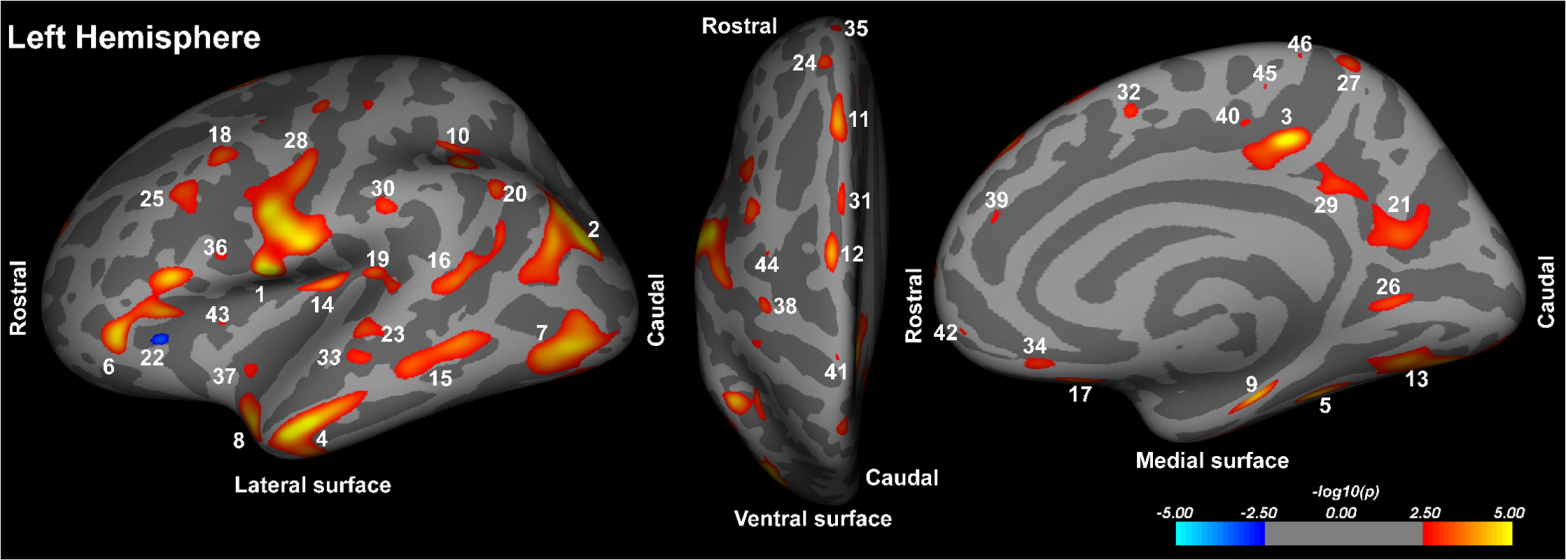
Left hemisphere brain regions with significantly reduced cortical thicknesses in HF over control subjects overlaid onto inflated pial surfaces. These sites with reduced cortical thickness included the precentral (1, 28, 41), inferior parietal (2, 20), posterior cingulate (3), middle temporal (4, 15, 33), fusiform (5, 13), pars triangularis (6, 22), lateral occipital (7), superior temporal (8, 19, 23), parahippocampal (9), supramarginal (10, 30), superior frontal (11, 12, 24, 31, 32, 35, 39), insula (14, 37, 43), tissue underlying superior temporal sulcus (16), medial orbitofrontal (17, 34, 42), caudal middle frontal (18, 25, 44), precuneus (21, 27), lingual (26), isthmus cingulate (29), pars opercularis (36), postcentral (38), and paracentral gyrus (40, 45, 46). The color scale represents false discovery rate-corrected p values less than 0.05 significance levels. Hot colors indicate reduced cortical thicknesses and cool colors show increased cortical thicknesses in HF compared to control subjects.

**Fig 2:**
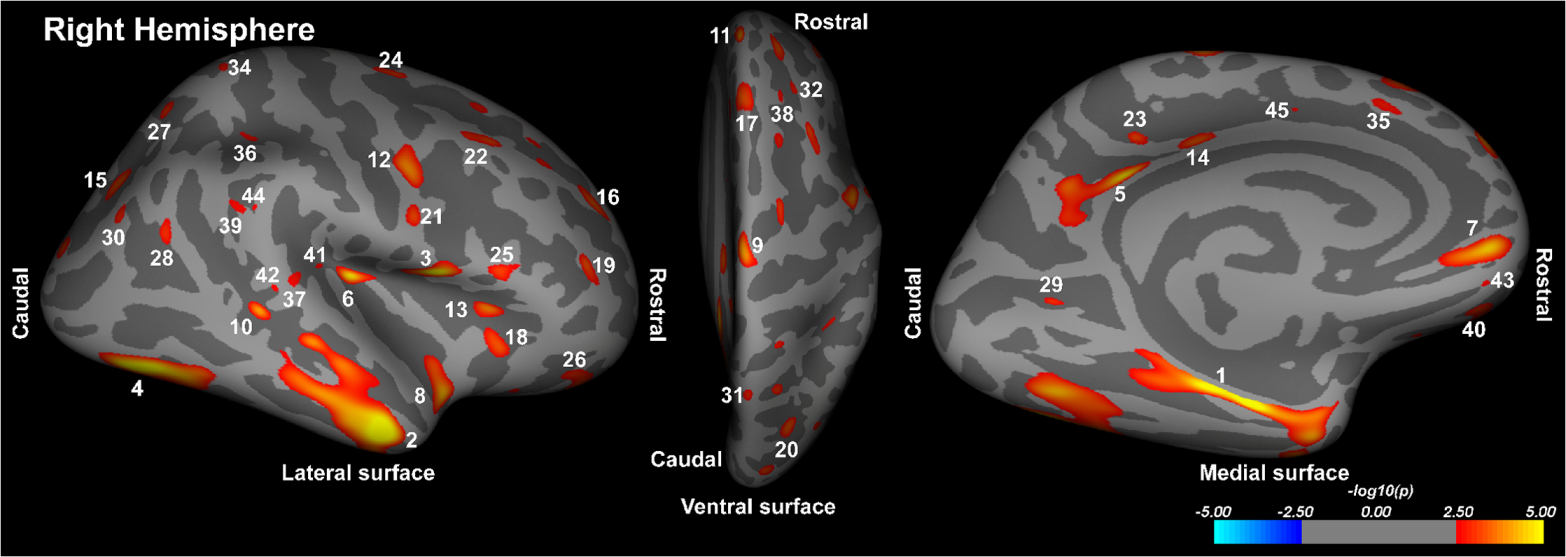
Right hemisphere brain areas with significantly reduced cortical thicknesses in HF compared to control subjects overlaid onto inflated pial surfaces. These cortical areas with changes included the entorhinal (1), middle temporal (2), precentral (3, 12, 21), fusiform (4), isthmus cingulate (5), insula (6), medial orbitofrontal (7, 43), superior temporal (8, 37, 41), paracentral (9), superior temporal sulcus (10, 42), superior frontal (11, 17, 24, 33, 35, 38, 45), pars triangularis (13), posterior cingulate (14, 23), superior parietal (15, 27, 31, 34), rostral middle frontal (16, 19, 32), lateral orbitofrontal (18, 26, 40), lateral occipital (20), caudal middle frontal (22), pars opercularis (25), inferior parietal (28, 30), lingual (29), and supramarginal (36, 39, 44). The color scale represents false discovery rate-corrected p values less than 0.05 significance levels. Hot colors indicate reduced cortical thicknesses and cool colors show increased cortical thicknesses in HF compared to control subjects.

HF patients showed significant cortical thinning in multiple areas, after controlling for age and gender (Tables 2 and 3; Figs 1 and 2). These areas with reduced cortical thicknesses appeared on both left and right hemispheres, including tissue underlying the superior temporal sulcus, caudal middle frontal, fusiform, inferior parietal, insula, isthmus cingulate, lateral occipital, lingual, medial orbitofrontal, middle temporal, para central, pars opercularis, pars triangularis, posterior cingulate, precentral, superior frontal, superior temporal, supra marginal (Tables 2 and 3 and Figs 1 and 2). Other unilateral cortical sites with cortical thinning in HF were the para hippocampal, postcentral, and precuneus on the left hemisphere (Table 2 and Fig 1), and entorhinal, lateral orbitofrontal, rostral middle frontal, and superior parietal on the right hemisphere (Table 3 and Fig 2), while the left pars triangularis showed increased cortical thickness in HF (Table 2 and Fig 1). Cortical thinning was more wide-spread in the orbitofrontal, precentral, mid temporal, and lateral occipital areas on the left side, and in the ventral-mid temporal and entorhinal cortices on the right side.

**Table 2:** Brain sites with significantly reduced cortical thicknesses in left hemisphere in HF over control subjects.

Brain sites	Max t statistic	Cluster size (mm^2^)	TalX	TalY	TalZ	Thickness (mm), mean ± SD	
						Controls	HF
Precentral	6.44	1293	-56.4	1.2	6.6	2.8±0.2	2.6±0.2
Inferior parietal	6.43	875	-36.9	-84.6	21.5	2.6±0.2	2.3±0.2
Posterior cingulate	5.43	210	-14.5	-34.1	38.1	2.3±0.2	2.1±0.2
Middle temporal	5.38	1025	-57.2	-7.7	-27.4	3.2±0.2	3.0±0.2
Fusiform	4.89	149	-32.1	-41	-22	3.1±0.2	2.9±0.2
Pars triangularis	4.79	593	-46.4	36.7	-7.3	2.6±0.2	2.4±0.2
Lateral occipital	4.74	794	-42.6	-75	-5.6	2.5±0.2	2.2±0.3
Superior temporal	4.70	295	-42.4	12.5	-27.1	3.6±0.3	3.4±0.3
Parahippocampal	4.53	71	-21.5	-23.5	-24.1	2.7±0.3	2.4±0.2
Supramarginal	4.43	311	-44.9	-50.1	40.8	2.4±0.2	2.2±0.3
Superior frontal	4.28	156	-10.3	42.3	45.1	3.0±0.2	2.8±0.2
Superior frontal	4.24	112	-17.2	5.2	65.3	2.9±0.2	2.7±0.3
Fusiform	4.21	1089	-28.8	-77.9	-8.5	2.3±0.2	2.1±0.2
Insula	3.77	168	-32.3	-23.5	13.8	2.5±0.2	2.3±0.2
Middle temporal	3.63	389	-61.4	-49	-3.4	3.1±0.3	2.8±0.3
Superior temporal sulcus	3.52	313	-52.3	-45.8	10	2.5±0.2	2.3±0.2
Medial orbitofrontal	3.43	63	-10.1	23.8	-17.7	2.3±0.2	2.2±0.2
Caudal middle frontal	3.32	129	-41.4	12.1	45.9	2.6±0.2	2.4±0.2
Superior temporal	3.32	138	-36.1	-35.5	11.9	2.3±0.2	2.1±0.3
Inferior parietal	3.29	79	-50.1	-61.2	34.3	2.6±0.3	2.4±0.2
Precuneus	3.27	318	-12.8	-59.6	20.4	2.6±0.2	2.4±0.2
Pars triangularis	-3.19	31	-34.1	32.7	-2.1	2.2±0.2	2.4±0.2
Superior temporal	3.15	126	-63.9	-21	-0.5	3.0±0.2	2.8±0.3
Superior frontal	3.11	58	-16.9	52.9	22.9	2.4±0.2	2.3±0.2
Caudal middle frontal	3.08	159	-43.5	20.7	34.6	2.4±0.2	2.3±0.2
Lingual	3.05	111	-20.4	-61.5	-0.3	1.7±0.3	1.5±0.2
Precuneus	3.04	67	-9.7	-51.4	68	2.5±0.2	2.3±0.2
Precentral	3.02	65	-40.8	-12.5	60.3	2.9±0.2	2.7±0.2
Isthmus cingulate	2.92	121	-10.4	-45.1	30.4	2.6±0.2	2.5±0.1
Supramarginal	2.88	52	-62.5	-30.4	28.7	2.9±0.3	2.7±0.2
Superior frontal	2.86	53	-9.9	20.7	59.1	3.0±0.2	2.8±0.2
Superior frontal	2.77	23	-8.6	7.4	51	2.7±0.2	2.6±0.2
Middle temporal	2.76	60	-48.6	-23.9	-11.4	2.4±0.2	2.2±0.3
Medial orbitofrontal	2.72	57	-4.5	32.5	-21.4	2.5±0.2	2.4±0.2
Superior frontal	2.59	28	-11	64.6	8.7	2.6±0.3	2.5±0.2
Pars opercularis	2.58	18	-45.4	9.3	16.7	2.5±0.2	2.4±0.2
Insula	2.52	21	-37.6	-8.9	-13.4	3.2±0.3	3.0±0.3
Postcentral	2.45	31	-41	-27.4	57.1	1.7±0.2	1.6±0.2
Superior frontal	2.34	8	-12.1	41.1	22	2.6±0.2	2.5±0.3
Paracentral	2.33	6	-16.3	-24.1	42.7	2.2±0.2	2.1±0.2
Precentral	2.31	5	-10.5	-27.3	71.9	2.7±0.2	2.6±0.2
Medial orbitofrontal	2.30	9	-7.2	50.5	-8.2	2.2±0.2	2.1±0.2
Insula	2.29	3	-30.1	12.5	6.4	2.6±0.2	2.5±0.1
Caudal middle frontal	2.28	4	-32.3	-1.3	46.8	2.5±0.2	2.4±0.2
Paracentral	2.27	1	-5.8	-30.2	52.2	2.6±0.2	2.4±0.2
Paracentral	2.26	3	-5.7	-41.6	63.8	2.2±0.2	2.0±0.2

Each brain area consists of adjacent voxels with significant group differences; some brain structures appear with multiple sites with significant changes. The magnitude of the peak (t statistic value) in each area and corresponding Tailarach coordinates (a standardized common brain space) are listed, together with cluster size (in normalized space) and mean and SD thickness values for control and heart failure subjects. Brain sites listed in table are with false discovery rate-corrected p values less than 0.05 significance levels.

**Table 3:** Brain areas with significantly reduced cortical thicknesses in HF from right hemisphere over control subjects.

Brain sites	Max t statistic	Size (mm^2^)	TalX	TalY	TalZ	Thickness (mm), mean ± SD	
						Controls	HF
Entorhinal	5.64	819	21.4	-14.1	-28	3.0±0.3	2.6±0.3
Middle temporal	5.59	1369	51.1	-5.7	-28.9	3.0±0.3	2.7±0.4
Precentral	5.37	251	48.5	6.6	3.2	2.8±0.2	2.6±0.2
Fusiform	5.23	1257	45.5	-67.5	-13.6	2.7±0.3	2.4±0.2
Isthmus cingulate	4.78	316	5.8	-41.5	31.2	2.8±0.2	2.6±0.2
Insula	4.76	103	31.9	-24.5	13.9	2.5±0.2	2.3±0.3
Medial orbitofrontal	4.53	247	11.5	48.9	-8.9	2.1±0.3	1.9±0.2
Superior temporal	4.29	206	42.3	-2.1	-20.7	3.1±0.3	2.8±0.3
Paracentral	4.08	128	6.4	-18	70	2.9±0.2	2.6±0.3
Superior temporal sulcus	4.07	52	44.4	-39.1	0	2.6±0.2	2.4±0.2
Superior frontal	4.05	74	7.7	56.1	29.5	2.9±0.1	2.8±0.2
Precentral	3.85	168	55.4	-1.9	39.6	2.8±0.2	2.6±0.3
Pars triangularis	3.79	54	33.4	25.1	9.7	2.2±0.2	2.1±0.2
Posterior cingulate	3.41	69	5.7	-21.6	39.7	2.8±0.2	2.7±0.3
Superior parietal	3.39	93	30.2	-64.8	25.4	2.2±0.2	2.0±0.2
Rostral middle frontal	3.37	107	25.8	44.1	31.6	2.4±0.2	2.3±0.2
Superior frontal	3.30	157	6.8	32.3	52.7	3.1±0.2	2.9±0.2
Lateral orbitofrontal	3.29	65	29.3	27.1	-3	2.7±0.2	2.5±0.3
Rostral middle frontal	3.26	86	39.2	44.6	13.4	2.2±0.2	2.1±0.2
Lateral occipital	3.21	94	22.9	-90.9	19	2.0±0.2	1.9±0.12
Precentral	3.18	49	58.9	6.2	26	2.9±0.2	2.8±0.2
Caudal middle frontal	3.16	114	34.1	20.8	47.7	2.7±0.2	2.5±0.2
Posterior cingulate	3.17	32	14.1	-37.7	39.6	2.3±0.2	2.2±0.2
Superior frontal	3.08	57	20.5	-5.8	57.9	2.6±0.2	2.4±0.3
Pars opercularis	3.08	78	54.8	22.8	15.8	2.7±0.2	2.5±0.2
Lateral orbitofrontal	3.08	156	19.4	42.6	-14.8	2.7±0.3	2.5±0.3
Superior parietal	3.00	38	19.9	-59.8	52.7	2.1±0.2	1.9±0.2
Inferior parietal	2.92	46	45.9	-60.3	21.4	2.5±0.2	2.3±0.3
Lingual	2.92	29	20.1	-66.7	2.1	1.6±0.2	1.5±0.1
Inferior parietal	2.86	46	40.5	-77.9	27.4	2.8±0.2	2.6±0.3
Superior parietal	2.82	37	9.7	-66.7	55.2	2.4±0.3	2.2±0.3
Rostral middle frontal	2.72	18	26.5	28.8	35.7	2.3±0.2	2.2±0.2
Superior frontal	2.71	28	21.8	14.1	49.8	2.5±0.2	2.3±0.2
Superior parietal	2.70	22	24	-47.4	61.1	2.2±0.2	2.0±0.2
Superior frontal	2.70	40	8.3	24	39.5	2.8±0.2	2.7±0.2
Supramarginal	2.70	25	35	-37.8	37.1	2.2±0.2	2.1±0.2
Superior temporal	2.60	36	62.4	-29.2	7.3	2.8±0.3	2.6±0.3
Superior frontal	2.60	16	22.5	27.1	40.9	2.4±0.2	2.2±0.2
Supramarginal	2.54	28	54.4	-44.2	26.7	2.6±0.3	2.4±0.3
Lateral orbitofrontal	2.52	14	23.5	23.7	-15.6	2.8±0.3	2.7±0.3
Superior temporal	2.51	11	39	-32.9	11.9	2.4±0.2	2.2±0.3
Bankssts	2.49	9	60.6	-33.3	5.1	2.7±0.3	2.5±0.3
Medial orbitofrontal	2.48	8	7.4	50.6	-20.9	2.7±0.3	2.5±0.2
Supramarginal	2.45	6	59.1	-40.7	27.3	3.0±0.3	2.8±0.3
Superiorfrontal	2.44	2	13	1	42.2	2.5±0.3	2.3±0.3

Each brain site consists of adjacent voxels showing a significant group differences; some brain areas appear multiple sites with significant differences. Table conventions are same as in Table 1.

### Relationships between regional cortical thicknesses and BMI

Although BMI significantly differed between groups, only limited brain sites showed significant relationships between BMI and regional cortical thicknesses in HF subjects. These sites with significant relationships with BMI included the pars triangularis (r = 0.4, p = 0.016) and middle temporal gyrus (r = -0.36, p = 0.033) on the left side, and precentral gyrus (r = -0.34, p = 0.046), superior frontal gyrus (r = -0.36, p = 0.033), and pars opercularis (r = -0.36, p = 0.032) on the right hemisphere.

## Discussion

### Overview

Multiple areas of the cerebral cortex in HF that play crucial roles in autonomic, cognitive, affective, language, and visual functions, showed reduced cortical thickness compared to control subjects. The laterality and location of the thinned regions reflect the spatial organization of cortical areas that mediate those deficient functions in HF. Thus, the right medial orbitofrontal cortex, which plays an important role in blood pressure regulation shows significant injury, while the analogous region on the left showed little damage. The affected cortical regions included temporal, frontal, occipital, and parietal areas, as well as cingulate and insular cortices that normally contribute substantially to deficient functions in the condition [7]. Although previous MRI studies showed lateralized structural changes in HF as altered brain tissue water diffusion, free water content, and loss of gray matter [4,6,7], and impaired functional responses to autonomic challenges in multiple gray and white matter areas [2,3], the present findings show precise localization of cortical tissue changes reflecting by reduced cortical thickness. The pathological processes contributing to the altered regional cortical thicknesses are unclear, but may include hypoxic/ischemic mechanisms resulting from low cardiac output, sleep-disordered breathing, diabetes, and hypertension in the condition, or initial damage in autonomic regulatory sites from mild stroke, maldevelopment, or infection, followed by secondary injury from impaired perfusion as a consequence of autonomic site injury. HF is accompanied by malabsorption, sodium restriction, and high fluid loss (typically, from diuresis) which can contribute to loss of water-soluble nutrients essential for neuronal and glial support.

Cortical thinning should not be unexpected, given the already demonstrated axonal injury in HF subjects, which showed significant loss of fibers and injury to supporting glia. Many of those axons represent projections derived from cortical neurons, and if injured, will be reflected as injury to those cortical neurons and supporting cells.

### Autonomic and motor regulation in HF

HF subjects show increased sympathetic tone and altered heart rate and blood pressure responses to blood pressure challenges, characteristics which are dependent upon appropriate functioning of right insular, cingulate, and right orbitofrontal cortices [2,3]; these areas showed reduced cortical thicknesses, indicating localized tissue injury in HF subjects. Although we did not examine autonomic control function in HF subjects, a subset of HF subjects included in this study showed autonomic control deficits in an earlier study [24]. The right and left insular cortices influence sympathetic and parasympathetic nervous system activity, respectively [25]; both left and right insulae showed decreased cortical thickness; however, the right insula showed more thinning, findings which are consistent with changes observed on previous MRI studies [2–4]. The cingulate cortex, which receives axons from and projects to insular cortices, mediates both autonomic branches, and damage to this structure can impact cardiac regulation [2,7]. The orbitofrontal cortex exerts prominent influences on somatomotor inhibition of autonomic responses, and coordination of behavioral responses during adaptation [26] and shows a substantial role in initiation of blood pressure responses [27]. Localized injury in this cortical area can contribute to a range of motor and autonomic deficits in the condition.

### Cognitive, affective, and dyspnea regulation in HF

HF subjects show various cognitive and affective issues and symptoms of dyspnea [7]. Along with other injured brain areas, including the hippocampus, anterior thalamus, fornix, mammillary bodies, caudate nuclei, putamen, cerebellum, described in previous studies [1,7], frontal cortices and para hippocampal areas are involved in cognitive, behavioral, and planning functions [28]. Frontal cortices send and receive information to various brain areas, including the caudate nuclei [29]. Behavioral and learning deficits result from lesioned frontal cortices, and similar deficits can be reproduced with caudate nuclei injury [29]. The para-hippocampal cortex projects to limbic areas, sites that are involved in higher-order cognitive and behavioral functions [30]. Thus, injury in frontal cortices and para hippocampal sites in HF may contribute to cognitive and behavioral issues, as shown here as well, in the syndrome.

HF patients show high levels of depressive symptoms and an increased incidence of mood disorders [31]; both mood and anxiety symptoms of HF subjects, included in this study, are significantly increased. Brain regions associated with mood regulation include the prefrontal cortex, para hippocampal gyrus, cingulate, insula, hippocampus, and cerebellum (not examined in this study) [7]. These brain sites are associated with injury in depressed subjects, and the majority of these areas showed reduced cortical thicknesses in HF subjects.

Signs of dyspnea are also a common characteristic of HF [7]. Dyspnea, the perception of breathlessness, is primarily regulated by the cingulate, insular, and cerebellar sites [32]. Both cingulate and insular areas showed reduced cortical thicknesses here, and may partially underlie the dyspnea commonly observed in the condition.

### Visual, language and speech regulation in HF

Abnormal visual, language, and speech functions are common in HF subjects. Multiple brain areas, including the superior temporal cortex, and tissue underlying the superior temporal sulcus, inferior parietal, lateral occipital, lingual, precuneus, superior parietal, and supra marginal cortical areas, which help regulate these deficient functions [33–36], showed reduced regional thicknesses in HF subjects. Superior temporal cortical areas that play significant roles in speech control [33], and tissue under the superior temporal sulcus and within inferior parietal regions that regulate language [34] show reduced cortical thicknesses in HF subjects. Other cortical sites that control visual domains include lateral occipital, lingual, precuneus, superior parietal, and supra marginal cortex [35,36]. The supra marginal gyri also play a crucial role in integration and interactions involving visual, auditory, and somato-sensory functions with adjoining sensory regions [36], Reduced cortical thicknesses in these visual and language regulatory areas may contribute to impaired behaviors.

Although cortical thickening appeared on both hemispheres, more wide-spread injury emerged in the precentral, mid temporal, and lateral occipital regions on the left side of the brain, and in the ventral-mid temporal and entrorhinal cortices on the right side. The left ventral precentral cortex is particularly involved in speech expression, with damage resulting in major motor difficulty in speech, an aspect which involves the tongue and other upper airway muscles. Mid temporal and entorhinal cortices are involved in memory and spatial cognition, and occipital areas in visual function. Although consequences of the lateralized injury are unclear, damage in these cortical sites may contribute to language [37], breathing [10], cognition [1], and visual issues found in the condition [38].

### Cortical thinning and potential for Alzheimer’s disease

Neuronal and glial changes or losses lead to cortical thinning, and such changes contribute to Alzheimer’s disease. Vascular dementia is accompanied by cortical thickness reduction as well, and regional cortical thinning found here in HF subjects poses a potential to contribute to symptoms of Alzheimer’s disease in the condition.

### Potential pathophysiology of reduced cortical thicknesses in HF

Various pathological processes may contribute to the regional changes in cortical thicknesses found here. These pathological processes include cerebral perfusion issues, resulting from low cardiac output [2], and hypoxia/ischemia processes, as a majority of HF subjects experience sleep-disordered breathing [10]; both aspects may contribute to the localized cortical changes. Other comorbid condition accompanying the condition, including Type 2 diabetes and hypertension, may also contribute to regional cortical thinning. Regional neural injury in various brain areas is reported in patients showing either condition in isolation. The cortical thinning may also result from earlier, pre-HF damage in autonomic control sites, including the insular, cingulate, and orbitofrontal cortices as described here, and the nucleus of the solitary tract, ventrolateral medullary, and cerebellar damage shown earlier [4,6]; this injury to autonomic regulatory areas may alter vascular supply to cortical regions, inducing secondary damage to the cortical regions. Stroke, developmental issues, or brain infections could also compromise vascular supply.

Micronutrient deficiencies, including low levels of thiamine and magnesium, are very common in HF subjects [39,40]. The frequent use of diuretics in HF can flush water-soluble nutrients, and HF subjects also show intestinal malabsorption [41], which can also contribute to thiamine and magnesium deficiencies [42,43]. Thiamine and magnesium are crucial components for carbohydrate metabolism, and when deficient, can diminish ATP generation necessary for cellular nutrition, resulting in cell death, a process accelerated by occasions of high energy demand, such as during hypoxia/ischemia [44,45]. Hypoxic/ischemic processes, along with low levels of thiamine and magnesium, can enhance brain tissue injury, resulting in reduced regional cortical thicknesses in the condition. Another instance of such nutritional deficiency is suspected in the profound reduction of mammillary body volumes in HF demonstrated elsewhere [7].

### Clinical implications

The findings reemphasize that HF is accompanied by seriously compromised brain tissue, as shown here as thinning in high-level brain areas, sites within the neocortex. These areas reflect both neuronal and other cell loss, and appear in specific regions that serve many of the autonomic, cognitive, and neuropsychological functions affected in the condition. Standard HF treatment does little or nothing to address those neural changes. However, it would be perhaps useful to develop strategies for neuroprotection found helpful in other “brain” conditions, such as stroke and neurodegenerative diseases. Those strategies might include interventions for defective blood brain barrier processes, known to be affected in HF [46], and specific efforts to assist glial support found valuable in Korsakoff’s syndrome and chronic alcoholism [47,48], and to minimize water-soluble nutrient loss through the very large fluid changes due to common use of diuretics in HF.

### Limitations

Limitations include selection of HF subjects with NYHA Functional Class II. We included HF subjects with relatively low severity (NYHA functional Class II), and excluded subjects with NYHA Functional Class III and IV. HF subjects with Functional Class III and IV are often unable to lay supine in the MRI scanner for significant periods of time, which restricted selection to Functional Class II patients only. Thus, these findings cannot be extended to NYHA Functional Classes III and IV. Use of diuretics is very common in HF subjects, and thus, hydration status may affect over-all brain volume and regional cortical thicknesses [49]. However, all HF subjects included in this study were hemodynamically-optimized, and were of stable weight, without significant use of diuretics, for at least six months prior to participation in the MRI study. We believe that the predominant regional cortical changes in this study result from chronic injury over acute hydration status in the condition. Other limitation of our study includes the cross-sectional nature of the study design; a longitudinal study could provide better insights into the nature of regional cortical thickness and show whether the injury may be reversible, which is less likely. Also, multiple cortical sites showed thinning in areas that regulate autonomic, neuropsychologic, and cognitive functions. Cognitive and neuropsychologic deficits are significant in HF subjects, included in this study, over control subjects, but we did not examine autonomic function in these HF subjects. However, the majority of the HF subjects of this study showed autonomic deficits, as shown in previously-published studies [2,3,24,49], and we believe that these HF subjects have autonomic abnormalities as well. A comparison of HF subjects with other subjects with prior equivalent medical histories may have been useful to determine HF-related neural changes. However, such subjects were not available to perform assessment. Since FreeSurfer procedures fail to parcelize cerebellar sites accurately, we did not include cerebellar cortices for regional assessment in thicknesses. Other limitations include the small sample size of HF subjects that precluded assessing clinically relevant subgroups among HF subjects, and the recruitment of all HF subjects from only one center, which may have yielded subjects with characteristics that differ from other populations.

## Conclusions

Lateralized cortical thinning emerged in HF compared to control subjects in multiple brain sites that regulate autonomic, cognitive, affective, language, and visual functions. The findings indicate chronic tissue alterations, with changes reflecting loss of neurons and glial cells in affected cortical sites. The pathological mechanisms contributing to reduced regional cortical thicknesses in HF likely include hypoxic/ischemic processes, resulting from impaired cerebral perfusion due to low cardiac output and sleep-disordered breathing, comorbid conditions, including diabetes and hypertension, or initial injury in autonomic regulatory sites from stroke, maldevelopment, or infection, leading to secondary damage to other brain regions. Injury also may be contributed by low levels of micronutrients, especially thiamine and magnesium, accompanying the condition.
